# Towards In-Vehicle Non-Contact Estimation of EDA-Based Arousal with LiDAR

**DOI:** 10.3390/s25237395

**Published:** 2025-12-04

**Authors:** Jonas Brandstetter, Eva-Maria Knoch, Frank Gauterin

**Affiliations:** 1 Institute for Vehicle Systems Engineering, Faculty of Mechanical Engineering, Karlsruhe Institute of Technology (KIT), 76131 Karlsruhe, Germany; eva-maria.knoch@kit.edu (E.-M.K.); frank.gauterin@kit.edu (F.G.); 2 Department of Complete Vehicle Data Driven Testing, Analysis and Vehicle Functions, Porsche Engineering Services GmbH, 74321 Bietigheim-Bissingen, Germany

**Keywords:** LiDAR, electrodermal activity (EDA), driver monitoring systems, arousal estimation, non-contact sensing, machine learning, deep learning

## Abstract

Driver monitoring systems are increasingly relying on physiological signals to assess cognitive and emotional states for improved safety and user experience. Electrodermal activity (EDA) is a particularly informative biomarker of arousal but is conventionally measured with skin-contact electrodes, limiting its applicability in vehicles. This work explores the feasibility of non-contact EDA estimation using Light Detection and Ranging (LiDAR) as a novel sensing modality. In a controlled laboratory setup, LiDAR reflection intensity from the forehead was recorded simultaneously with conventional finger-based EDA. Both classification and regression tasks were performed as follows: feature-based machine learning models (e.g., Random Forest and Extra Trees) and sequence-based deep learning models (e.g., CNN, LSTM, and TCN) were evaluated. Results demonstrate that LiDAR signals capture arousal-related changes, with the best regression model (Temporal Convolutional Network) achieving a mean absolute error of 14.6 on the normalized arousal factor scale (–50 to +50) and a correlation of r = 0.85 with ground-truth EDA. While random split validations yielded high accuracy, performance under leave-one-subject-out evaluation highlighted challenges in cross-subject generalization. The algorithms themselves were not the primary research focus but served to establish feasibility of the approach. These findings provide the first proof-of-concept that LiDAR can remotely estimate EDA-based arousal without direct skin contact, addressing a central limitation of current driver monitoring systems. Future research should focus on larger datasets, multimodal integration, and real-world driving validation to advance LiDAR towards practical in-vehicle deployment.

## 1. Introduction

Affective computing is an interdisciplinary field of research that deals with the development of systems that can recognize, interpret, and simulate emotional states. In the context of vehicles, the focus lies on detecting the emotional and cognitive states of drivers, with the aim of improving traffic safety, optimizing interaction with driver assistance systems, and enabling personalized driving experiences.

A major focus is safety, with the goal of detecting safety-critical states such as fatigue, stress, or distraction at an early stage and controlling driver assistance systems in an adaptive manner accordingly. Through the continuous monitoring of cognitive and emotional states, reductions in attention or adverse emotional strain can be detected. Furthermore, state-adaptive vehicle control can be implemented to adjust vehicle behavior to the driver’s psychophysiological state, for example by modifying warning strategies or intervention timings. In addition, a better understanding of the driver allows the in-vehicle experience to be personalized through affect-adaptive systems, such as by adjusting infotainment or climate control to the emotional state.

The growing relevance and interest in affective computing is also reflected in the increase of more than 70% in scientific publications on the topic between 2017 and 2023, according to Pei et al. [1]. This rise in research activity is primarily attributed to technological maturity and increasing application demands. Recent advances in remote physiological sensing illustrate a similar trend, where non-contact optical and imaging-based methods have attracted growing attention across different research communities [2]. A recent study has emphasized the continued growth of research on in vehicle affective and cognitive state estimation and highlighted the increasing relevance of non-contact sensing approaches in this context [3]. Furthermore, global trends such as human–machine symbiosis create the need to equip machines with emotional intelligence in order to enable more interactions.

Affective computing relies, on the one hand, on information derived from speech, including intonation, speaking rate, and prosody. On the other hand, input is also obtained from visual cues such as facial expressions, gestures, and body posture, as well as from physiological signals, including electroencephalography (EEG), electromyography (EMG), EDA, heart rate, and heart rate variability (HRV).

EDA, also referred to as galvanic skin response (GSR) or skin conductance (SC), is of particularly high informational value. Its direct dependencies allow machine learning (ML) approaches to achieve accuracies well above 90% when mapping EDA values to affective states, as summarized by Sánchez-Reolid et al. [4]. EDA also provides objective data. Its basis is skin conductance, which is influenced by the activity of eccrine sweat glands. This objectivity holds across languages, cultures, and interpretative frameworks. In contrast to questionnaires or verbal statements, EDA is non-interpretative. It delivers direct, measurable data that are not affected by subjective judgments or linguistic encoding. Another advantage is that EDA occurs largely unconsciously and is therefore minimally, if at all, subject to voluntary control. Consequently, even when individuals attempt to conceal their emotions, EDA frequently reveals their arousal states. Furthermore, latent or subliminal states can be detected through EDA. These include states that are not consciously perceived, remain hidden or unapparent, or occur below the threshold of conscious awareness. Foundational dermatological studies have shown how near-infrared light interacts with hydration levels, sweat gland activity, and subsurface scattering layers of the skin, which provides a physiological explanation for reflectivity changes induced by sympathetic activation [5].

EDA is typically characterized by two parameters: skin conductance level (SCL) and skin conductance response (SCR). SCL represents the tonic (slow) component of EDA, which primarily reflects the general arousal state of the sympathetic nervous system. SCR is the phasic component, indicating rapid responses to stimuli and therefore representing the primary focus of affective computing research. In other words, if the SCR, the phasic component, can be measured, it becomes possible to make a near real-time assessment of the subject’s affective state.

Classical EDA measurement is based on determining skin conductance or skin resistance, both of which vary with the activity level of the sweat glands. A small direct current or direct voltage is applied between two electrodes, with measurements typically taken at the fingers or palms. The recorded values are either conductance, expressed in microsiemens (µS), or resistance, expressed in kiloohms (kΩ) [6,7].

This conventional, contact-based measurement method is unproblematic in the majority of application scenarios and is therefore widely used in psychophysiological research, clinical psychology, psychiatry for diagnostic and therapeutic support, forensic applications, as well as educational and learning research [8,9].

However, if one wishes to measure EDA values from the driver or passengers in a vehicle during driving, the conventional method is almost impossible to implement, as it requires contact with skin surfaces. The majority of published studies employ contact-based, often prototype, measurement technology [10,11,12,13,14,15].

In isolated cases, investigations have been conducted that use the steering wheel, as the only directly available skin contact surface with the vehicle, as a measurement point. For example, Ye et al. [16] developed a Smart Steering Sleeve *S*^3^ incorporating a Sweat Sensing Unit (SSU), which enables real-time EDA measurement. However, significantly more studies have focused on electrocardiogram (ECG) electrode measurements, which, in principle, could be adapted for use in EDA measurement. Such investigations were carried out, among others, by Warnecke et al. [17] and Babusiak et al. [18]. Nevertheless, these approaches remain challenging in terms of cost–benefit ratio and packaging, and are not yet integrated into series production.

Accordingly, the use of contactless measurement methods becomes a compelling alternative. In particular, near-infrared distance sensing offers advantages over camera-based approaches since its signal is largely independent of visible illumination and does not record identifiable imagery. At the same time, it provides consistent geometric information that can be used to observe surface reflectivity which makes LiDAR an attractive candidate for in cabin physiological monitoring. This is consistent with recent LiDAR-based person detection research that highlights stable geometric observations and robustness to illumination changes in dynamic environments [19]. Nevertheless, visual approaches based on RGB imaging remain scarcely explored, with only limited procedures documented in the literature. Bhamborae et al. [20,21] demonstrate that EDA can be recorded in a contactless manner via brightness changes in RGB videos (LumEDA), achieving high correlations with sensor-based measurements, particularly in response to auditory and physiological stimuli. The study was conducted under laboratory conditions, with the palms of the hands observed in the video. Likewise, a study by Uchida et al. [22] observes the palms of the hands under laboratory conditions and attempts to correlate the “wetness” of the skin and thus sweating with associated emotion analysis. In principle, these approaches are subject to extreme noise and interference, mainly resulting from changing lighting conditions and movement. Beyond optical imaging, radar-based approaches have demonstrated robust non-contact estimation of respiration, heart rate, and subtle human motion and therefore constitute an important reference modality for contactless physiological monitoring [23,24].

Thus, it is reasonable, on the one hand, to select other body sites than the palmar surfaces as the observed body part, and, on the other hand, to consider a medium other than light in the visible spectrum. Regarding the first point, i.e., other suitable body sites for observation, answers can be found in the literature. Hossain et al. [25] investigated the positions forehead, neck, finger, and foot, and concluded that the sole of the foot is the best alternative to finger-based EDA. The forehead is also robust against artifacts, but less sensitive and subject to inter-individual variability. Van Dooren et al. [26] arrived at a similar conclusion after investigating 16 different body sites that could be used for the detection of emotion-induced sweating. The highest SC reactivity was found for foot, finger, shoulder, and forehead. Kamei et al. [27] also conducted their experiments at the forehead, stating that the forehead is suitable for the observation of continuous, basal sweat production and for general arousal.

The forehead is therefore very suitable as a position in the in-vehicle scenario, as it can be easily observed. In this work, LiDAR is used as the sensing modality. A LiDAR system is an active optical measurement method that determines the distance to objects by measuring the time of flight of emitted laser pulses and the reflected light. In addition to distance, it typically also records the intensity of the backscattered signal, which enables conclusions about the reflective properties of the surface, such as material, color, or roughness. Most LiDAR systems operate at near-infrared wavelengths, typically around 850 nm, 905 nm, or 1550 nm, depending on application requirements and sensor design.

LiDAR systems for perception outside the vehicle are widespread and widely adopted. LiDAR systems for the vehicle interior have been intensively researched and tested for some time and are on the verge of being introduced into series-production vehicles. In a first step, such systems will probably be used for occupant monitoring (Occupant Monitoring Systems, OMS) and for passenger counting and classification, as well as for attention and drowsiness detection, gesture control, and interaction with the human–machine interface (HMI). Most existing approaches rely on radar or camera-based systems. Automotive studies have also demonstrated that Doppler radar systems can reliably detect respiration and heart rate in non-contact in cabin scenarios, illustrating the practical relevance of radar-based physiological monitoring [28]. For example, Gharamohammadi et al. [29] proposed a volume-based occupancy detection method achieving high accuracy in distinguishing adults, infants, and empty seats using radar sensing, while Mishra et al. [30] presented an in-cabin monitoring framework leveraging cameras and artificial intelligence (AI) for safety and human–machine interaction. In contrast, the potential of LiDAR for in-cabin applications has not yet been extensively explored, although its robustness to lighting conditions and high spatial resolution make it a promising candidate for future occupant monitoring systems. At the same time, LiDAR is increasingly being investigated in research projects and early prototypes, indicating a growing interest in bringing this technology into practical in-vehicle use.

Three-dimensional LiDAR has been widely explored for human detection and tracking, leveraging dense point clouds for robust recognition. Gómez et al. [31] demonstrated efficient 3D LiDAR-based human detection and tracking in indoor environments, achieving reliable performance across different poses. Similarly, Jia et al. [19] showed that 3D LiDAR significantly outperforms 2D LiDAR for person detection, offering higher accuracy and robustness in complex scenes. These results highlight the potential of 3D LiDAR for precise localization of body parts, including the head and forehead region, in in-cabin monitoring applications.

This paper addresses the central question of whether EDA values can be approximated based on LiDAR reflection behavior. To explore this, an initial experiment is conducted using a simple setup. The approximation of EDA-related states is implemented using methods from AI. Both feature-based classification and sequence-based classification approaches are investigated. Finally, the paper discusses the associated challenges and limitations, and outlines directions for future work.

## 2. Methodology—Materials and Methods

The aim is to investigate the fundamental feasibility of contactless approximation of EDA values using LiDAR data. For this purpose, a simplified, stationary experimental setup is developed, which captures the required LiDAR reflection intensity with a single LiDAR measurement point. The ground-truth data are collected using a conventional finger-based EDA sensor. Data synchronization is carried out via a compact microcomputer. The stimulus presentation is performed visually through an app displaying images from the Open Affective Standardized Image Set (OASIS) [32], with the aim of deliberately evoking arousal responses in the participants. The measured EDA data are prepared in a preprocessing step. Subsequently, model approaches based onv feature-based classification and sequence-based classification are described.

### 2.1. Participants and Experimental Protocol

**Figure 1:**
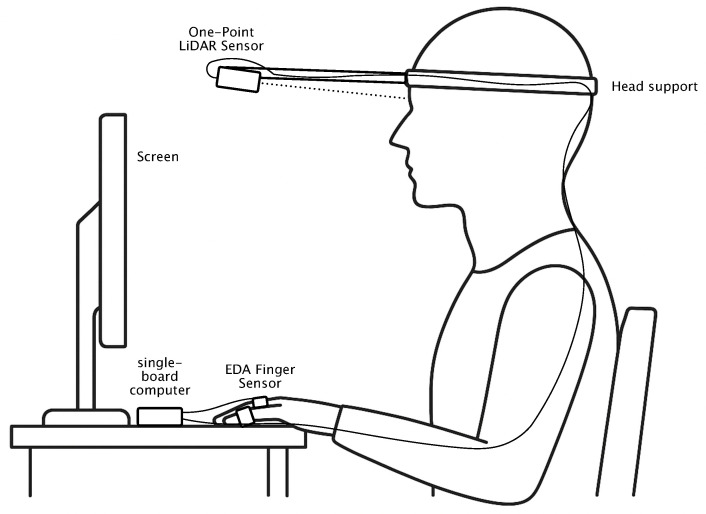
Prototypical experimental setup. A single-point LiDAR sensor is mounted on a head frame to capture forehead reflections. In parallel, contact-based EDA is recorded from the fingers. Visual stimulation is provided via images displayed on the screen. Illustration created with the assistance of OpenAI GPT-5.

The experiment is conducted under laboratory conditions with the aim of minimizing environmental variables, disturbances, and motion artifacts as much as possible. The experimental setup is illustrated in Figure 1. This is achieved by having the participant seated at a table with a screen in front, on which the stimuli for stimulation are displayed. The participant is fitted with a head-mounted frame to which a single-point LiDAR sensor is affixed. The sensor is aligned to a fixed position on the forehead, thereby almost eliminating distance and motion artifacts.

#### 2.1.1. Sensor Technology: LiDAR Sensor

A time of flight LiDAR module of the type TFmini S from Benewake in Beijing in China was used as the sensor [33]. The sensor provides both distance and intensity values at a sampling rate of up to 100 Hz. Due to its small size and narrow field of view (approximately 2–3°), it is particularly suitable for spot-wise acquisition of reflection intensity on the forehead surface. As the sensor is compact and lightweight, it can be mounted centrally and perpendicularly in front of the forehead on a head mount. This allows for a fixed alignment at a distance of 15–17 cm and thus a minimal angular deviation to the forehead. In the present work, only the reflection intensity (signal strength) was used for further processing. The sensor’s design makes it appropriate for exploratory laboratory studies, but it is not calibrated for absolute intensity measurements. Data were transmitted through a serial UART interface to the central microcomputer, which was a Raspberry Pi 5 from the Raspberry Pi Foundation in Cambridge in the UK [34].

#### 2.1.2. Sensor Technology: EDA Sensor

For the acquisition of the EDA data, a GSR sensor module that is functionally equivalent to the Grove GSR from Seeed Studio in Shenzhen in China was used [35]. The module measures the skin-conductance-dependent electrical resistance via two electrodes attached to the fingers. The resulting analog voltage output was digitized via an ADC converter for further processing and recorded synchronously with the LiDAR data using the microcomputer. The electrodes were attached to the non-dominant hand of the participant to minimize movement-related disturbances. In this study, the sensor served as the ground-truth reference for the evaluation of the LiDAR-based EDA approximation. Due to its open design and limited filtering, the sensor is primarily suitable for exploratory studies in laboratory settings. Disturbance factors such as skin moisture, movement, or temperature fluctuations can influence the signal and were minimized through controlled conditions.

#### 2.1.3. Design of Stimulus Presentation

The aim of the stimulus presentation is to evoke emotional responses, which serve as the basis for introducing variation in the ground-truth data. For stimulation, images from the OASIS dataset are shown [32]. The procedure is as follows: the participant sits at a table and is equipped with the EDA sensor on the hand and the LiDAR system on the head mount, as shown in Figure 1. A dark screen is presented on the monitor in front of the participant for the first 5 min to serve as an initial resting-state measurement. During this period, the participant calms down, and the SCL can be determined for later removal in the postprocessing stage. After the 5 min have elapsed, a random image from the OASIS set is shown for 5 s each. In total, 240 images are shown, selected from the 900 images available in the full dataset. The start and end of data recording are automatically controlled by the start and end of the app run.

#### 2.1.4. System Control and Data Acquisition

A Raspberry Pi 5 [34] was used as the central control and acquisition unit. It was responsible both for stimulus presentation via a custom-developed application and for synchronous recording of the LiDAR and EDA data via serial interfaces. The data were stored locally and subsequently exported for analysis. No further processing was carried out on the device.

### 2.2. Data Processing and Feature Extraction

**Figure 2:**
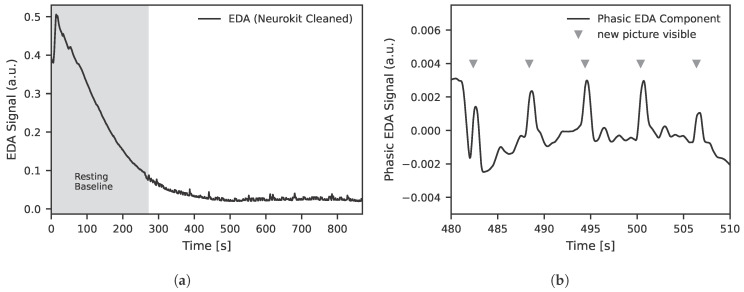
The diagrams present original EDA data from two different subjects that serve as the basis for the model training: (**a**) illustrates the EDA signal across the entire experiment after preprocessing with the NeuroKit2 toolbox [36]. The EDA signal is reported in arbitrary units (a.u.) due to normalization steps applied by NeuroKit2 to enhance comparability across participants. The observed decline in the EDA signal over time reflects a typical habituation response, characterized by reduced sympathetic arousal during prolonged resting conditions. (**b**) displays a segment during the experiment, where the phasic EDA response to each newly presented image or stimulus is clearly visible.

The aim is to synchronize and clean the raw signals so that they can be used as input for training the models. This also includes extracting the relevant phasic component from the EDA signal. The data from the EDA sensor are sampled at 20 Hz. Subsequently, the raw data are cleaned using the NeuroKit2 toolbox [36]. The complete course of the EDA signal after cleaning for one experimental run is shown in Figure 2a. A detailed excerpt of the measurement during the presentation of images from the OASIS dataset is shown in Figure 2b.

**Figure 3:**
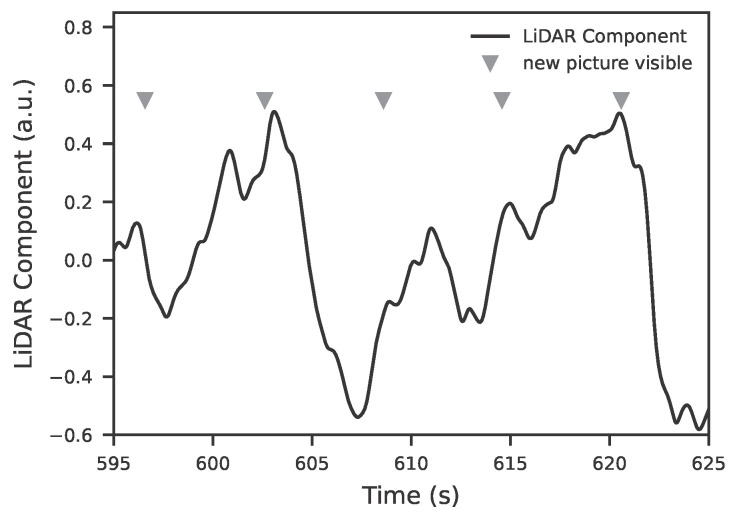
LiDAR signal strength data after filtering with the NeuroKit2 toolbox. The LiDAR signal is reported in arbitrary units (a.u.) due to normalization steps applied by NeuroKit2 to enhance comparability across participants. The depicted segment shows real measurement data together with the time points at which a new image/stimulus was presented to the participant.

The LiDAR data are sampled at 20 Hz, analogously to the EDA data. From the LiDAR system, only the reflection intensity (signal strength) is used. The cleaning is also carried out analogously to the EDA data using the NeuroKit2 toolbox [36]. This means that the LiDAR signal strength data are treated in the same way as the EDA data and preprocessed accordingly, as shown in Figure 3. Both signals were processed with identical cleaning steps using the NeuroKit2 toolbox. This included low pass filtering to remove high frequency noise, detrending procedures to address slow baseline drift and a decomposition of phasic and tonic components for the EDA signal. All signals were then z-normalised on a per participant basis to compensate for subject specific amplitude differences. These preprocessing steps ensured consistent scaling and comparability across modalities and subjects.

**Figure 4:**
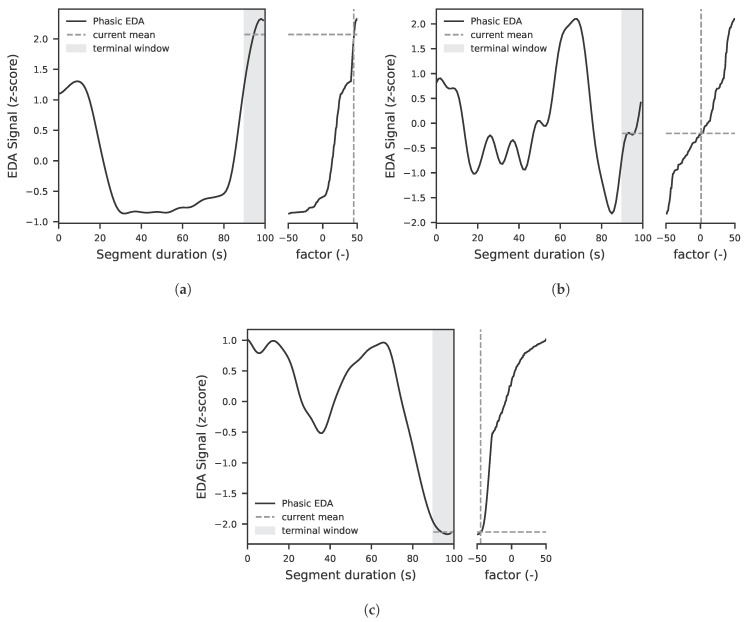
The figures illustrate the methodology used to determine the relative factor. The observation window spans 100 s. Within this period, the phasic EDA value is z-normalized. The left part of each diagram shows the signal of the EDA, while the smaller diagram on the right displays the sorted rank progression. The dashed horizontal and vertical lines indicate the position of the mean EDA from the last 10 s within the rank diagram; this mean determines the position of the vertical line and corresponds to the calculated factor. This rank value is then shifted by an offset of −50 to simplify interpretation. (**a**) shows the case of a strongly positive increase, (**b**) a neutral case, and (**c**) a strongly negative one.

To capture the phasic response, each EDA segment is first z-normalized. Subsequently, the current value, averaged over a very short segment at the end of the window, is positioned relative to the distribution of all segment values by determining its empirical rank percentile. The rank percentile reflects the relative position of the current value within the segment distribution, where higher percentiles indicate that the current activity exceeds most preceding values and thus marks stronger phasic activation. This percentile indicates how many of the segment values are less than or equal to the current value, with respect to the entire segment. The result is a normalized scale value between 0 (minimum) and 100 (maximum). By subtracting a constant offset of 50, the value is symmetrically centered, such that 0 corresponds to a neutral course, +50 to a strongly positive, and –50 to a strongly negative arousal change. Figure 4 illustrates this for a strongly positive (a), a neutral (b), and a strongly negative (c) factor. This continuous factor represents the reaction dynamics and is robust against inter-individual differences in absolute signal amplitude. With this value, it is now possible to train models on a single, meaningful metric. This also allows for compensating the extreme inter-individual differences in EDA values and responses among individuals. Rather than comparing absolute values, only a relative value is considered, which is referenced to a preceding segment. All models were trained on one hundred second segments of the LiDAR and EDA signals. Segments were extracted in a sliding manner without overlap unless otherwise stated. The terminal window corresponds to the last ten seconds of each segment and its mean value was used as the current value for the percentile-based factor estimation. The choice of a non overlapping sliding scheme ensured that each segment represented an independent observation while keeping temporal adjacency between consecutive windows.

### 2.3. Modeling Approach

In this study, the classification and regression of a factor derived from the phasic electrodermal activity (EDA) signal, as described in Section 2.2, are investigated. A feature-based pipeline that operates on time-series features using established machine-learning methods, and a sequence-based pipeline that learns directly from the LiDAR time series using deep neural networks. The goal of this work is not to propose new architectures but to assess feasibility using standard Python toolboxes. All experiments were conducted using Python version 3.13.0.

For classification, the continuous factor is discretized into *k*-balanced classes within a range from −50 up to +50. For regression, the factor is predicted as a continuous target in [0, 100] (reported on the centered scale [−50, +50] for agreement analyses). Unless stated otherwise, models are trained with a 75/25 random split of segments. Classification performance is reported using balanced accuracy (BA) and normalized improvement (NI), while regression is evaluated using mean absolute error (MAE), root mean squared error (RMSE), and the coefficient of determination (*R*^2^). Detailed descriptions of the feature-based and sequence-based classification and regression approaches are provided in Section 2.3.1–Section 2.3.4.

#### 2.3.1. Feature-Based Classification Approach

For each segment, statistical and time-based features were extracted from the preprocessed and cleaned LiDAR signal. Automatic feature computation was performed using the Python library catch22 [37] in version 0.4.5. The target variable to be learned was derived from the phasic EDA factor and divided into *k* equally sized classes (range −50 to +50). Classification was performed using established models from the scikit-learn library, as listed in Table 1. Two evaluation strategies were employed to assess model performance. The first was Leave-One-Subject-Out Cross-Validation (LOSO), which evaluates generalizability to previously unseen subjects. The second involved a randomized split of the entire dataset into training and test sets, independent of subject identity, serving as a comparative scenario that represents the theoretical upper bound of model performance.

#### 2.3.2. Sequence-Based Classification Approach

Recurrent and convolutional architectures implemented in PyTorch 2.7.1 were used for sequence-based classification [38]. The models employed in this study are listed in Table 1. The input consisted of segmented LiDAR intensity profiles (single channel, sampling rate: 20 Hz) with a segment length of 10 s. The target variable comprised k discrete trend classes (ranging from strongly negative to strongly positive), derived from the phasic EDA component (Section 2.2). All models consisted of a feature extraction layer (GRU/LSTM or CNN/TCN) followed by a fully connected output layer. Adam was used as the optimization algorithm with a learning rate of 0.001, in combination with cross-entropy loss. Training was conducted for 75 epochs with a batch size of 32. To assess generalization performance, two validation strategies, random split and LOSO, were employed, analogous to the feature-based classification approach.

#### 2.3.3. Feature-Based Regression Approach

In addition to classification, we performed a complementary regression analysis to directly predict the continuous factor derived from the phasic EDA factor (Section 2.2). For each segment, statistical and time-series features were computed from the LiDAR signal with the package catch22 [37]. The implementation used scikit-learn [39], and the models are shown in Table 2. Predictions were clipped to the valid range [0, 100] and reported on the centered scale (−50…+50) for agreement analyses. Implementation used scikit-learn.

#### 2.3.4. Sequence-Based Regression Approach

We also trained sequence models as regressors operating directly on the LiDAR time series analog to classes with the models shown in Table 2. Architectures followed Section 2.3.2, with the classification output replaced by a single linear unit. The models were optimized using mean squared error (MSE) as the loss function and trained with the Adam optimizer (learning rate = 0.001), a batch size of 128, and 75 epochs. As for feature regression, predictions were clipped to [0, 100] and reported on the centered scale (−50…+50). We report results for a 75/25 random split to match the feature-based setting.

## 3. Results

**Figure 5:**
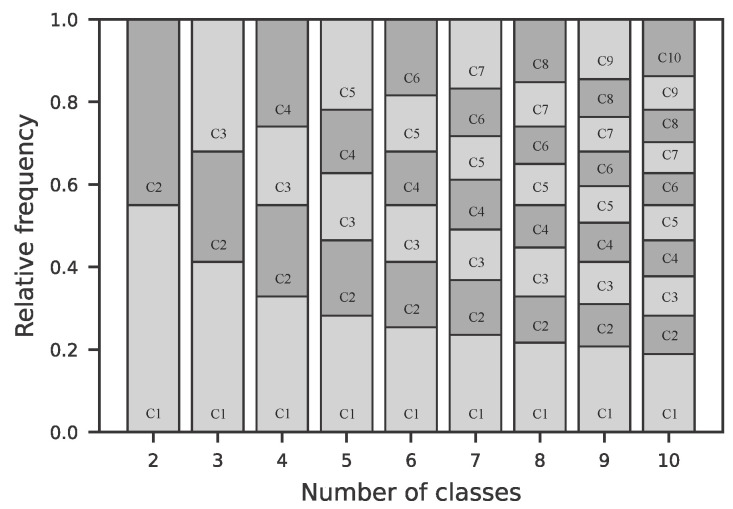
Stacked bar plots illustrate the relative class distributions for configurations with two to ten classes. Different shades of gray indicate different classes, labeled from C1 to C10. Exact imbalance ratios are summarized in Table 3.

The dataset collected using the experimental setup described in Section 2.1 includes five test subjects, three female and two male. A total of 62,634 instances for classification were recorded. The models were trained on varying numbers of target classes. The classes are uniformly distributed within the range of −50 to +50. The calculation of this factor is described in Section 2.2. The distribution across individual classes for each class count is shown numerically in Table 3 and visually in Figure 5. The class imbalance across the different configurations varies, with an imbalance ratio between 1.2 and 2.5, which can be considered a moderate imbalance [40].

**Figure 6:**
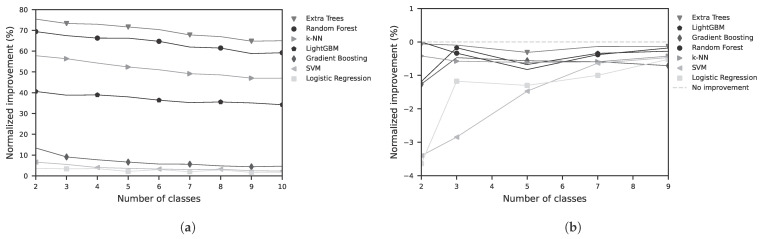
Normalized improvement of various feature-based models as a function of the number of classes to be learned. (**a**) validation is performed using a random split with 75% for training and 25% for testing; (**b**) Validation is performed using the LOSO approach. The normalized improvement is computed relative to the expected accuracy of random guessing, which corresponds to a uniform probability of correct classification (e.g., 50% for a two-class problem).

The results of the feature-based models are shown in Figure 6a for validation using a random split, and in Figure 6b for validation using the LOSO approach. The result diagrams depict the normalized improvement (NI) as a function of the number of target classes to be classified. The normalized improvement is calculated according to Equation (1), where *c* denotes the chance level (c=1/k) for *k* classes.

(1) $$\begin{array}{c} NI=\frac{BA-c}{1-c} \end{array}$$

**Figure 7:**
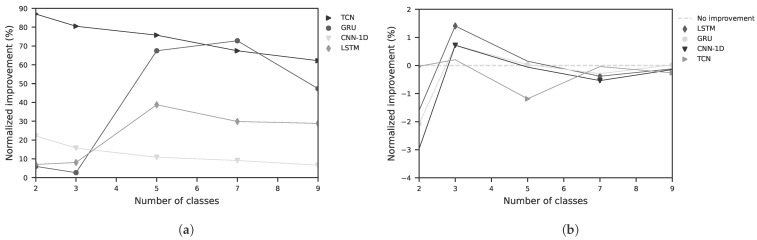
Normalized improvement of various sequence-based (deep learning) models as a function of the number of classes to be learned. (**a**) validation is performed using a random split with 75% for training and 25% for testing; (**b**) Validation is performed using the LOSO approach.

The results of the sequence-based classification using deep learning are shown for the different classes in the random split setting in Figure 7a and in the LOSO setting in Figure 7b.

**Figure 8:**
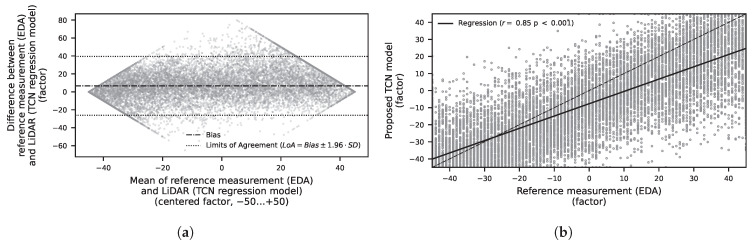
Visualization of the results of the TCN regression models. (**a**) Bland–Altman plot comparing the approximated EDA factor obtained with the proposed LiDAR method against the ground-truth EDA measurement. Each dot represents an individual paired measurement (difference versus mean). The solid line indicates the mean bias, while the dotted lines represent the 95% limits of agreement (LoA = Bias ± 1.96 · SD). The mean is 6.67 (−) and the standard deviation (SD) is 16.75 (−). The diamond-shaped pattern visible in the plot results from the limited range and discrete distribution of the approximated EDA factor values. (**b**) Scatter plot of the EDA factor estimated with the proposed LiDAR-based method versus the ground-truth EDA measurement. Each dot represents an individual paired measurement. The dashed line indicates the line of identity (y = x), while the solid line shows the linear regression fit (r = 0.85, *p* < 0.001).

Detailed results for a three-class problem are presented in Table A1 and Table A2. For classical feature-based models, as shown in Figure 6, validated using random split, an Extra Trees classification model yields the best performance with a BA of 82.2%. In contrast, when validated using LOSO, the BA drops to 33.3%. This results in a normalized improvement (NI) of +73.3% for random split validation and −0.1% for LOSO, as shown in Table A2. The regression results for the feature-based models are presented in Table A3. The best performance was achieved by a Random Forest Regressor with a mean absolute error (MAE) of 16.8 (−) and a root mean square error (RMSE) of 19.7 (−). The unit corresponds to the introduced factor ranging from –50 to +50. The coefficient of determination *R*^2^ was 0.61. The results for the sequence-based models are shown in Table A4. Here, the best performance was obtained with a Temporal Convolutional Network (TCN), yielding an MAE of 14.6, an RMSE of 18.8, and an *R*^2^ of 0.65. In addition to the MAE and RMSE values, a Bland–Altman analysis was performed to evaluate the agreement between the proposed LiDAR method and the contact-based EDA measurement as ground truth. Figure 8 shows the differences plotted against the mean of the two measurements. The mean bias was 6.67, and the 95% limits of agreement (LoA = Bias ± 1.96 · SD) ranged from –10.1 to 23.4. Most data points fell within these limits, indicating good agreement. In addition to the Bland–Altman analysis, a scatter plot of the estimated EDA factor versus the ground-truth values (Figure 8) was generated. The analysis yielded a Pearson correlation coefficient of *r* = 0.85 (*p* < 0.001), indicating a weak but statistically significant linear association between the two methods.

## 4. Discussion

This study demonstrates the feasibility of estimating EDA-based arousal in an in-vehicle setting using a non-contact LiDAR approach. Data were obtained in a controlled table-top setup under standardized stimulus conditions, representing a first step towards in-vehicle deployment. Both feature-based and sequence-based classification models achieved promising accuracy, indicating that LiDAR signals contain sufficient information to capture arousal-related physiological changes. These findings provide initial evidence that remote EDA estimation is possible without direct skin contact, thus addressing an important limitation of conventional sensor-based driver monitoring systems.

These results are consistent with previous work on remote physiological sensing, where non-contact methods such as video- and radar-based approaches have been shown to capture autonomic activity [20,21,22]. Compared to camera-based systems, LiDAR offers inherent robustness against lighting variations and potential privacy concerns, which makes it particularly attractive for in-vehicle deployment. In addition, LiDAR does not rely on ambient illumination and therefore provides stable measurements under strong or rapidly changing lighting, which is a common condition in real driving environments. In addition, LiDAR captures geometric depth information with higher spatial precision than short range radar, which supports reliable localization of small facial regions without recording identifiable visual data. These properties underline its potential as a privacy-preserving and robust sensing modality for cabin monitoring. Furthermore, while radar-based approaches have demonstrated the ability to track respiration and heart rate [41,42,43], our findings suggest that LiDAR can also extend remote monitoring to electrodermal dynamics, thereby complementing existing modalities. Radar-based systems already play an important role in non-contact driver monitoring and have demonstrated reliable estimation of respiration, heart rate, and micro-motion under real world conditions. Their performance is robust across larger distances and they are well established in automotive sensing. However, radar typically provides limited spatial resolution, which makes precise localization of facial areas more challenging. LiDAR offers complementary strengths since it delivers stable geometric information and is largely unaffected by visible illumination. This allows LiDAR to focus on reflectivity-based surface changes at specific regions of interest such as the forehead which may carry arousal related physiological information. As a result, radar and LiDAR should be considered complementary rather than competing modalities.

The physiological link between electrodermal activity and near-infrared reflectivity can be explained by the effect of sympathetic activation on sweat gland filling, hydration, and superficial skin moisture [5]. Increases in phasic EDA are accompanied by subtle changes in the optical properties of the stratum corneum, including variations in absorption and scattering that influence the intensity of reflected near-infrared light. Controlled studies in skin optics have shown that hydration and sweat duct dynamics affect reflectivity at near-infrared wavelengths. These mechanisms provide a plausible basis for the observed relationship between LiDAR intensity and EDA and motivate future work that integrates multiple physiological markers to obtain a more comprehensive representation of stress and arousal.

The implications of these findings are twofold. First, they demonstrate the potential of LiDAR-based sensing as a foundation for next-generation driver monitoring systems that move beyond conventional contact sensors. By enabling remote estimation of arousal, LiDAR could contribute to more adaptive and emotionally intelligent vehicles, ultimately enhancing safety and user experience. Second, the results highlight that non-contact physiological monitoring can be seamlessly integrated into existing automotive sensor infrastructures, thereby facilitating practical deployment without requiring additional intrusive hardware.

An important observation in this study is the discrepancy between classification performance obtained with random splits and with LOSO validation. While random splits yielded relatively high accuracies, performance decreased substantially under LOSO evaluation. This highlights the challenge of achieving robust generalization across individuals, a key requirement for practical in-vehicle deployment. The results suggest that subject-specific patterns in LiDAR-based EDA estimation can be learned effectively, but that inter-individual variability remains a major factor. Addressing this variability through larger datasets, transfer learning, or personalized model adaptation represents an important avenue for future research.

Beyond algorithmic performance, practical deployment in vehicles poses additional challenges. In particular, successful LiDAR-based EDA estimation requires that the subject’s forehead is reliably detected and captured within the three-dimensional cabin point cloud. Variations in seating position, head orientation, or occlusions caused by accessories (e.g., hats, glasses) can hinder consistent signal acquisition. This emphasizes the need for robust occupant detection and tracking algorithms that ensure stable forehead localization, which is a prerequisite for translating the proposed approach into real-world driving environments.

Despite these promising results, several limitations need to be acknowledged. The study was conducted with a relatively small sample size under controlled laboratory-like driving conditions, which may limit the generalizability to real-world scenarios. In addition, the focus was restricted to EDA-based arousal, whereas multimodal integration with other physiological signals might provide a more comprehensive understanding of driver states. Moreover, the machine learning models employed in this study were not specifically optimized or tailored to the task. This design choice reflects the emphasis on demonstrating feasibility rather than maximizing performance. Future work should therefore investigate larger datasets, more diverse contexts, and optimized model architectures to fully exploit the potential of LiDAR-based arousal estimation.

Building on these initial findings, future work should focus on validating the proposed LiDAR-based approach in naturalistic driving environments and across larger, more diverse populations. Real-time implementation and optimization of the classification models will be crucial to assess the feasibility of on-board deployment in intelligent vehicles. Furthermore, combining LiDAR with complementary modalities, such as radar, cameras, or steering behavior, may enable multimodal driver state monitoring that is both robust and unobtrusive. Ultimately, this line of research paves the way towards emotionally aware vehicles capable of supporting safer and more personalized human–machine interaction.

The small sample size represents an important limitation of the current study. With only five participants, the results cannot be generalized to a broader population and subject specific reflectivity patterns may have had a strong influence on the model behavior. This is also reflected in the discrepancy between random split and LOSO evaluations. Larger and more diverse datasets will therefore be essential to address demographic variability and physiological differences and to reduce the risk of statistical bias in the learned models. The present work should be regarded as a first proof of concept which provides preliminary evidence for the feasibility of LiDAR-based arousal estimation, but future studies need to validate the approach under more realistic and heterogeneous conditions.

In addition to the limited sample size, the experimental setup relied on a fully fixed head position and stationary environmental conditions which do not reflect realistic in vehicle variability. Real driving involves continuous changes in posture, viewing direction and distance to the sensing device and these factors can influence the stability of the reflected near-infrared signal. It will therefore be important to investigate to what extent LiDAR-based arousal estimation remains robust when natural head movements and dynamic cabin conditions are present.

A further limitation concerns the evaluation methodology. The high performance observed in the random split scenario indicates that the models captured consistent within subject patterns, but this also implies a potential risk of overfitting since segments from the same participant appear in both training and test sets. The substantially lower performance in the leave-one-subject-out setting highlights that generalization across individuals remains a major challenge. This suggests that future work should focus on larger and more heterogeneous datasets and explore approaches such as subject independent feature extraction, domain adaptation, or personalized model calibration.

## 5. Conclusions

This work provides a first proof-of-concept demonstration that LiDAR can be used for non-contact estimation of EDA-based arousal in a vehicle-related context. Using a controlled table-top setup, both feature-based and sequence-based classification and regression models were able to capture arousal-related physiological information from LiDAR signals, thereby addressing a central limitation of conventional contact-based driver monitoring. The results highlight both the potential and the challenges of LiDAR-based approaches: while feasibility was confirmed, generalization across individuals and practical requirements such as reliable forehead detection remain open issues. Nevertheless, the findings underline the promise of LiDAR as an integral component of future multimodal, non-intrusive driver monitoring systems. With continued research on robustness, real-time implementation, and multimodal integration, LiDAR has the potential to support the development of emotionally aware vehicles that enhance both safety and user experience.

## Figures and Tables

**Table 1:** Table of the classification models used for feature-based and sequence-based approaches. The feature-based models were implemented using the scikit-learn toolbox scikit-learn 1.7.0, while the sequence-based models were implemented with PyTorch 2.7.1 [38].

Feature-Based Classifiers	Sequence-Based Classifiers
Extra Trees	Gated Recurrent Unit (GRU)
Random Forest	Long Short-Term Memory (LSTM)
k-Nearest Neighbors (k-NN)	1D Convolutional Neural Network (1D-CNN)
Light GBM	Temporal Convolutional Network (TCN)
Gradient Boosting	
Support Vector Machine (SVM)	
Logistic Regression	

**Table 2:** Table of the regressor models used for feature-based and sequence-based approaches. The feature-based models were implemented using the scikit-learn toolbox [39], while the sequence-based models were implemented with PyTorch 2.7.1 [38].

Feature-Based Regressors	Sequence-Based Regressors
Extra Trees Regressor	Gated Recurrent Unit (GRU)
Random Forest Regressor	Long Short-Term Memory (LSTM)
LightGBM Regressor	1D Convolutional Neural Network (CNN-1D)
Gradient Boosting Regressor	Temporal Convolutional Network (TCN)
XGBoost Regressor	

**Table 3:** Imbalance ratio for different configurations of the number of target classes. An imbalance ratio of 1 indicates perfect balance. Higher values denote increasing class imbalance. Figure 5 shows the distribution across the different class configurations.

	Number of Classes								
	2	3	4	5	6	7	8	9	10
Imbalance Ratio	1.22	1.54	1.73	1.84	1.96	2.23	2.39	2.48	2.51

## Data Availability

The data presented in this study are available on request from the corresponding author.

## Abbreviations

The following abbreviations are used in this manuscript:

BA	balanced accuracy
CNN	convolutional neural network
EDA	electrodermal activity
EEG	electroencephalography
EMG	electromyography
GRU	gated recurrent unit
GSR	galvanic skin response
HMI	human–machine interface
HRV	heart rate variability
LiDAR	Light Detection and Ranging
LOSO	leave-one-subject-out
LSTM	long short-term memory
MAE	mean absolute error
NI	normalized improvement
OMS	occupant monitoring system
R^2^	coefficient of determination
RMSE	root mean squared error
SC	skin conductance
SCL	skin conductance level
SCR	skin conductance response
SVM	support vector machine
TCN	temporal convolutional network

## Appendix A

**Table A1:** Results for the three-class classification problem. For the feature-based models, both the balanced accuracy and the normalized improvement are reported. Bold values indicate the best performing model within each row.

		Feature-Based Classification (Three Classes)						
		Extra Trees (%)	Random Forest (%)	k-NN (%)	Light GBM (%)	Gradient Boosting (%)	SVM (%)	Logistic Regression (%)
BA	random split	**82.2**	78.3	70.9	59.2	39.4	37.0	35.6
	LOSO	**33.3**	33.1	33.0	33.2	33.0	31.4	32.6
NI	random split	**+73.3**	+67.5	+56.4	+38.8	+9.1	+5.5	+3.4
	LOSO	**−0.1**	−0.8	−0.6	−0.2	−0.5	−2.9	−1.5

BA: balanced accuracy, NI: normalized improvement, LOSO: leave-one-subject-out.

**Table A2:** Results for the three-class classification problem. For the sequence-based models, both the balanced accuracy and the normalized improvement are reported. Bold values indicate the best performing model within each row.

		Sequence-Based Classification (Three Classes)			
		GRU (%)	LSTM (%)	CNN-1D (%)	TCN (%)
BA	random split	35.1	38.7	43.8	**87.0**
	LOSO	33.8	**34.3**	33.8	33.5
NI	random split	+2.6	+8.1	+15.7	**+80.5**
	LOSO	+0.7	**+1.4**	+0.7	+0.3

**Table A3:** Results for feature-based regression models. Bold values indicate the best performing model within each row.

	Extra Trees	Random Forest	LGBM Regressor	Gradient Boosting	XGB Regressor
MAE (−)	20.386	**16.820**	18.579	26.200	18.016
RMSE (−)	23.577	**19.733**	21.833	29.967	21.321
$R^{2}$ (−)	0.446	**0.612**	0.525	0.105	0.547

**Table A4:** Results for sequence-based regression models. Bold values indicate the best performing model within each row.

	GRU	LSTM	CNN-1D	TCN
MAE (−)	27.943	27.941	27.301	**14.588**
RMSE (−)	31.672	31.672	31.108	**18.779**
$R^{2}$ (−)	0.000	0.000	0.035	**0.648**
